# Novel nano-semiconductor film layer supported nano-Pd Complex Nanostructured Catalyst Pd/Ⓕ-MeO_x_/AC for High Efficient Selective Hydrogenation of Phenol to Cyclohexanone

**DOI:** 10.1038/s41598-017-01255-9

**Published:** 2017-04-28

**Authors:** Jiaqi Si, Wenbing Ouyang, Yanji Zhang, Wentao Xu, Jicheng Zhou

**Affiliations:** Key Laboratory of Green Catalysis and Chemical Reaction Engineering of Hunan Province, School of Chemical Engineering Xiangtan University, Xiangtan, 411105 Hunan Province China

## Abstract

Supported metal as a type of heterogeneous catalysts are the most widely used in industrial processes. High dispersion of the metal particles of supported catalyst is a key factor in determining the performance of such catalysts. Here we report a novel catalyst Pd/Ⓕ-MeO_x_/AC with complex nanostructured, Pd nanoparticles supported on the platelike nano-semiconductor film/activated carbon, prepared by the photocatalytic reduction method, which exhibited high efficient catalytic performance for selective hydrogenation of phenol to cyclohexanone. Conversion of phenol achieved up to more than 99% with a lower mole ratio (0.5%) of active components Pd and phenol within 2 h at 70 °C. The synergistic effect of metal nanoparticles and nano-semiconductors support layer and the greatly increasing of contact interface of nano-metal-semiconductors may be responsible for the high efficiency. This work provides a clear demonstration that complex nanostructured catalysts with nano-metal and nano-semiconductor film layer supported on high specific surface AC can yield enhanced catalytic activity and can afford promising approach for developing new supported catalyst.

## Introduction

Catalytic hydrogenation of phenol to cyclohexanone is a reaction of commercial and environmental significance, for the hydrogenation product is an important raw material for caprolactam and adipic acid, which are the monomer of nylon-6 and nylon-66, respectively^1^. Generally, phenol can be hydrogenation to cyclohexanone in a “one-step” or a “two-step” process. In two-step, phenol is hydrogenated to cyclohexanol firstly and cyclohexanol is dehydrogenated to yield cyclohexanone subsequently^2, 3^. The one-step process avoids the endothermic step of dehydrogenation and therefore is certainly preferable in terms of capital costs and energy savings. Selective directly hydrogenation of phenol to cyclohexanone conducts either in gas or liquid phase in the literatures^4–7^. In general, the vapor phase hydrogenation must experiences harsh conditions such as high pressure and temperature, which usually causes catalyst deactivation by coking, generating undesirable by-products and complicating the separation^8, 9^. Hence liquid phase hydrogenation can be great interest because of the relatively mild reaction conditions^10, 11^. Many researchers have contributed to this area, and multiple catalysts have been screened, such as Pd/C^12^, Pd/Al-MCM-41, Ru/Al-MCM-41^13^, Pd/MHSS, PdAu/MSHH^14, 15^, Pd/MIL-101^16^. However, achieving a high selectivity (95%) at elevated conversion (80%) under mild conditions is a great challenge. Notably, Han and his co-worker^17^ reported that a complete phenol conversion with >99.9% selectivity to cyclohexanone could be achieved on a dual-supported Pd-Lewis acid catalyst in sc-CO_2_, while the conditions is so sophisticated (supercritical carbon dioxide as solvent, which requires high H_2_ and CO_2_ pressures >7.0 MPa). Most recently, Wang and co-works designed a kind of carbon nitride material as support to prepare Pd-based catalysts^18^, which achieved both excellent conversion and selectivity in the hydrogenation of phenol in aqueous media. Nevertheless, the preparation of the mpg-C_3_N_4_ and/or CN-x was complicated and involved the use of NH_4_HF_2_ or HF which are hazardous. On the other hand, Chen and co-works utilized polymer-fuctionalized CNF and/or ionic liquid-like copolymer to stabilize Pd^19–21^, and the as-prepared catalysts have been proven to be efficient for phenol hydrogenation. However, the preparation of these catalysts required many expensive polymers and had the disadvantage of high preparation cost. In this regard, a high efficient selective phenol hydrogenation over on facile catalysts is quiet desirable.

It is generally accepted that the nature of the catalysts support has a considerable impact on the product composition. Scirè *et al*.^22^ compared the catalytic performance of Pd/La_2_O_3_, Pd/CeO_2_ and Pd/Al_2_O_3_ for phenol hydrogenation, and they found that the order of active and selectivity to cyclohexanone was the following: Pd/La_2_O_3_ >Pd/CeO_2_ >Pd/Al_2_O_3_, which is reverse with the order of acidity, in terms of strength and number of acid sites. Corma *et al*.^23^ studied the effect of acid-base properties of supports on the phenol hydrogenation. They found that phenol tended to absorb on the basic support, such as MgO, through the hydroxyl group which gave more cyclohexanone. When the support was acidic, such as Al_2_O_3_, phenol tended to absorb on the support through aromatic ring and would get more cyclohexanol. Therefore, controlled the nature of support is the key issue to design the Pd-cased for phenol hydrogenation.

Many industrial catalysts consist of expensive metals dispersed on inexpensive high-area porous supports. The high cost of noble metals has limited their industrial applications, and the utilization efficiency of noble metals in conventional supported catalysts is far less than satisfactory. Research has been reported that when the dispersions are high, many of the metal atoms are present at a surface, accessible to reactants and available for catalysis. Flytzani-Stephanopoulos *et al*.^24–26^ showed that atomically dispersed Au or Pt cations exhibited excellent catalytic activity for water-gas shift reaction, while Au or Pt particles had no chemical action. Another solution of the significantly reducing the use of precious metal, preparation of support single-atom catalysts (SAC), was proposed by Zhang and coworkers^27–29^. Our group came up with a better strategy to utilize noble metal efficiently, and prepared a novel complex nanostructured catalyst by photocatalytic reduction method^30–32^. Gold nanoparticles were finely dispersed on the nano-TiO_2_ films, which was tiled on the mesoporous materials MCM-41 or MCM-22. The as-prepared catalysts exhibits fantastic activity and chemical stability for cyclohexane oxidation.

Herein, we present a novel semiconductor nano-film layer supported Pd nanoparticles complex nanostructured catalyst Pd/Ⓕ-MeO_x_/AC for high efficient selective hydrogenation of phenol to cyclohexanone. The complex nanostructured catalyst was prepared by a facile method: nano-semiconductor (TiO_2_ or CeO_2_) film layer was spread on the high specific surface support (AC) and then Pd nanoparticles were anchored on the nano-semicondutor film layer/AC by photocatalytic reduction method. Furthermore, it is demonstrated that the highly efficient catalytic behavior of Pd/Ⓕ-TiO_2_/AC toward phenol selective hydrogenation could be attributed to the synergistic effect between Pd nanoparticles and nano-semiconductor (TiO_2_ or CeO_2_) and their complex nanostructured properties.

## Results and Discussion

The textural of the samples were investigated by using N_2_ adsorption-desorption measurements (Figure S1). It is suggested that all the samples is mesoporous following a type IV adsorption isotherm with an important hysteresis loop. Table S1 exhibits the surface area and pore parameters of the prepared catalyst samples. It is noted that the BET surface area of TiO_2_/AC decreased slightly after loading with Pd nanoparticles, but still had a considerable value of 1138 m^2^/g.

**Figure 1:**
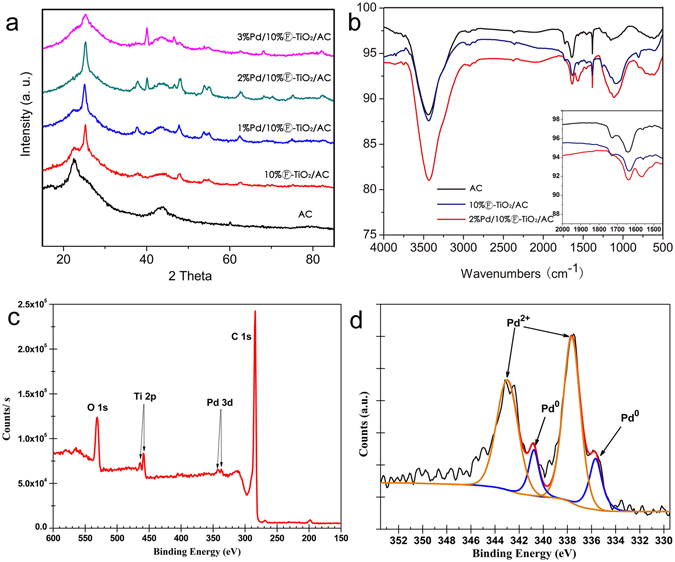
(**a**) XRD patterns of the prepared AC, 10% Ⓕ-TiO﻿_2_﻿/AC, and 1–3%Pd/Ⓕ-TiO_2_/AC. (**b**) FT-IR spectra of AC, 10%ⒻTiO_2_/AC and 2%Pd/10%Ⓕ-TiO_2_/AC. (**c**) XPS scan survey for Pd/Ⓕ-TiO_2_/AC catalyst. (**d**) Pd 3d XPS spectra of Pd/Ⓕ-TiO_2_/AC catalyst.

The formation of Pd nanoparticles was proven by powder XRD (Fig. 1a). All samples present broadened peaks at 22.6° and 43.3°, which could be attributed to the AC support. It can be observed that 10%Ⓕ-TiO_2_/AC and 1–3%Pd/10%Ⓕ-TiO_2_/AC exhibit several diffraction peaks at 25.3°, 37.8°, 48.0°, 53.9°, 55.1°, 62.7°, 68.8°, 70.3°, 75.0°, corresponding to (101), (004), (200), (105), (211), (204), (116), (220), and (215) planes of anatase phase of TiO_2_ (JCPDS 21–1272)^33^. Two addition diffraction peaks at 40.1° and 46.7° could be assigned to the (111) and (200) planes of Pd metal particles in the samples of Pd/10%Ⓕ-TiO_2_/AC^34^. It is clear to see that the (111) plane of Pd gets sharper, as the Pd content increased.

Figure 1b shows the FT-IR spectra of the AC, 10%Ⓕ-TiO_2_/AC and 2%Pd/10%Ⓕ-TiO_2_/AC samples, which presents the vibrations of the various groups at different wavenumbers. For all three samples, a broad peak at 3440 cm^−1^ can be assigned to the stretching vibration of surface-bound O-H groups. A new band at around 1060 cm^−1^ is observable in 10%Ⓕ-TiO_2_/AC and 2%Pd/10%Ⓕ-TiO_2_/AC, which can be ascribed to Ti-O-C, indicating probably the presence of the interaction between TiO_2_ and AC^35^. Also, the Ti-O-C band gets stronger in 2%Pd/10%Ⓕ-TiO_2_/AC. In addition, AC spectra show two peaks in the region from 1600 to 1700 cm^−1^ identified to C=O group. The region shows a clear red shift when Pd nanoparticles are anchored on the 10%Ⓕ-TiO_2_/AC. These changes in FT-IR spectra seem to suggest that an interaction between Pd nanoparticles and Ⓕ-TiO_2_/AC have formed. According to Loh’s report^36^, a coordination between Pd:O might have formed, that is, the electron pair of O occupy the vacant orbital of Pd. The formed interaction could be an important reason for the high efficient phenol hydrogenation in the presence of Pd/Ⓕ-TiO_2_/AC catalyst.

Figure 1c and d display the X-ray photoelectronic profiles of survey and Pd 3d spectra of the 2%Pd/10%Ⓕ-TiO_2_/AC catalyst, respectively. As known in Fig. 1d, the Pd presents a doublet corresponding to Pd 3d_5/2_ and Pd 3d_3/2_. The Pd 3d_3/2_ peak at 340.8 eV and the Pd 3d_5/2_ peak at 335.6 eV are attributed to Pd^0^, while the peaks at 337.5 eV (Pd 3d_3/2_) and 342.5 eV (Pd 3d_5/2_) can be assigned to Pd^+^^2^.

**Figure 2:**
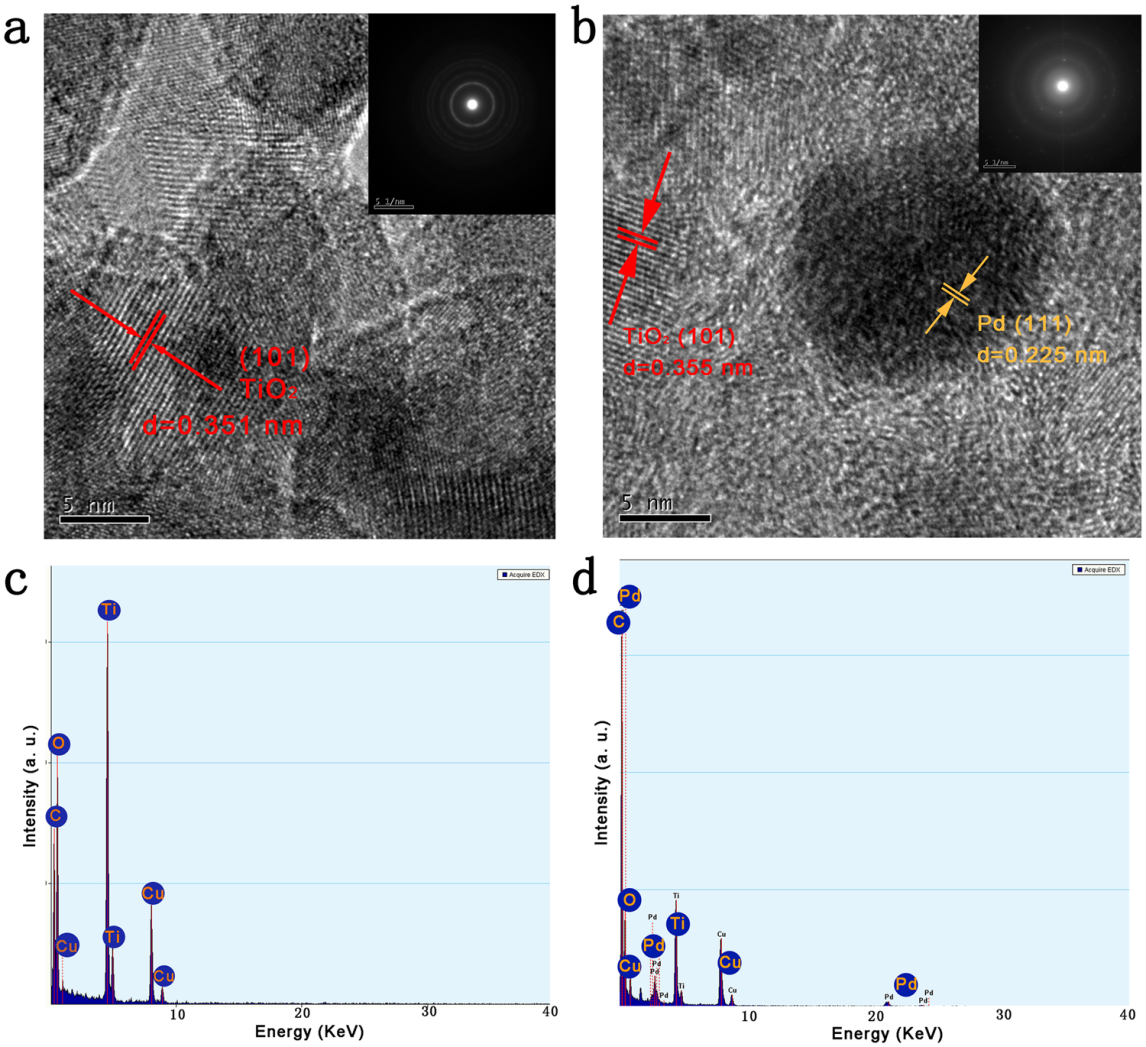
(**a**) HRTEM image of 10%Ⓕ-TiO_2_/AC. (**b**) HRTEM image of 2%Pd/10%Ⓕ-TiO_2_/AC. The insets of (**a** and **b**) are the corresponding SAEM images. (**c** and **d**) are EDX analysis of 10%Ⓕ-TiO_2_/AC and 2%Pd/10%Ⓕ-TiO_2_/AC, respectively.

**Figure 3:**
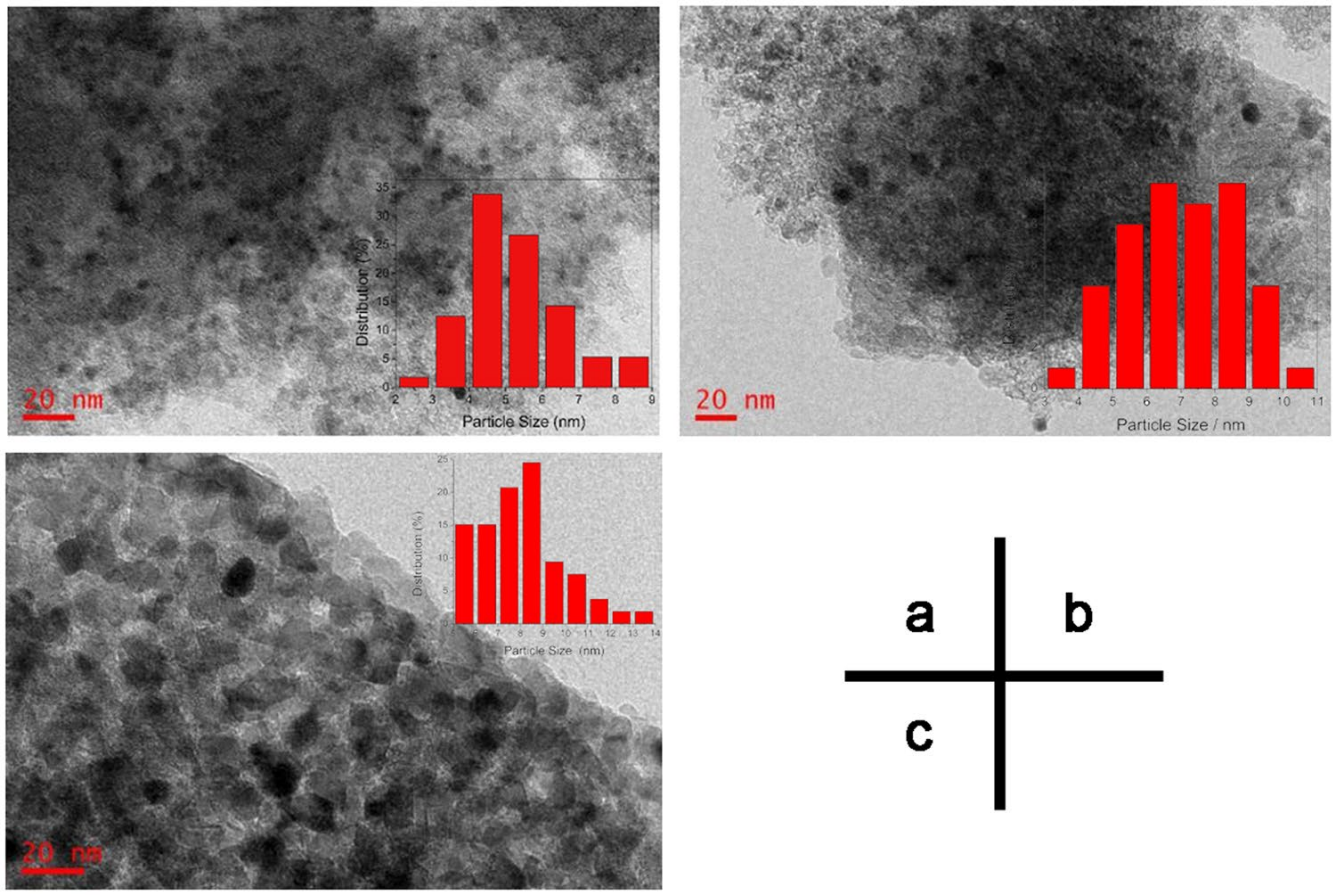
TEM images and Pd nanoparticles size distributions of 1%Pd/10%Ⓕ-TiO_2_/AC (**a**), 2%Pd/10%Ⓕ-TiO_2_/AC (**b**), 3%Pd/10%Ⓕ-TiO_2_/AC (**c**).

HRTEM micrographs of 10%Ⓕ-TiO_2_/AC and 2%Pd/10%Ⓕ-TiO_2_/AC are shown in Fig. 2. Proper lattice spacing of the (101) growth direction of anatase TiO_2_ and the (111) growth direction of Pd nanoparticles are clearly seen in the HRTEM micrographs, which are corroborating the XRD results. The selected area electron diffraction (SAED) patterns of the samples are shown in the insets of the HRTEM images. These images indicate characteristic circles origination from the corresponding crystal planes of anatase TiO_2_ and Pd nanoparticles. It is noteworthy that only nano TiO_2_ can be observed in the HRTEM image of 10%Ⓕ-TiO_2_/AC, which indicated that nano-TiO_2_ had coated on the activated carbon surface. Figure 3(c) and (d) show the EDX data of 10%Ⓕ-TiO_2_/AC and 2%Pd/10%Ⓕ-TiO_2_/AC. The data indicate the presence of elemental constituents within the as-synthesized samples.

Figure 3 shows the TEM images of the Pd/Ⓕ-TiO_2_/AC samples. The palladium nanoparticles are evident as small dark spots, while the Ⓕ-TiO_2_/AC substrate is brighter and larger. It can be seen that the palladium nanoparticles are fairly homogeneously distributed over the surface of the Ⓕ-TiO_2_/AC. As can be seen in the histograms, an increase in the contents of Pd causes an increase in the particle size of the Pd nanoparticles. According to principles of the spontaneous monolayer distribution, 10%Ⓕ-TiO_2_/AC could construct a platelike semiconductor film supported on AC, thus the as-prepared catalyst Pd/10%Ⓕ-TiO_2_/AC could form a complex nanostructured composed of nano-semiconductor film layer and Pd nanoparticles.

**Figure 4:**
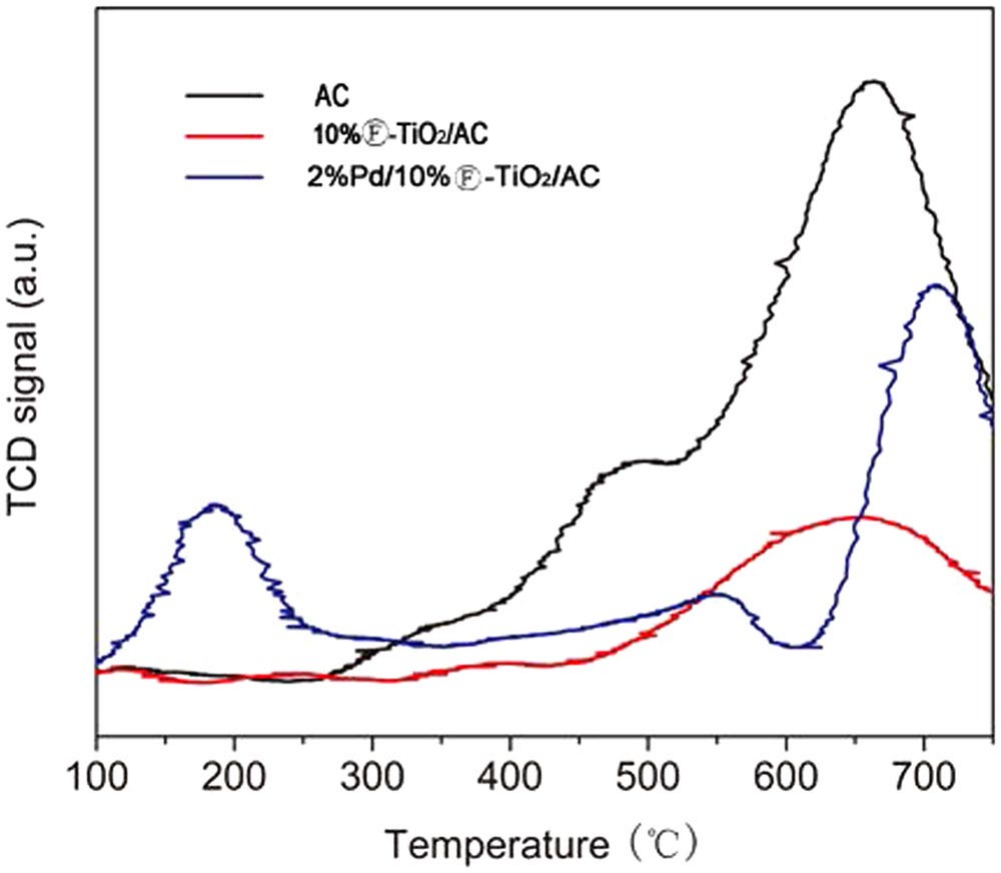
The H_2_-TPR profiles of AC, 10%Ⓕ-TiO_2_/AC and 2%Pd/10%Ⓕ-TiO_2_/AC.

H_2_-TPR experiments were performed to further confirm the Pd nanoparticles and the support as shown in Fig. 4. Two positive peaks at 480 °C and 650 °C in the AC sample could be attributed to the oxygen-containing functional groups like -COOH, -OH,=O, etc.^37^. The broad peak observed between 350 °C and 450 °C was due to the reduction of TiO_2_ in the 10%Ⓕ-TiO_2_/AC sample. The reduction of Ti^4+^ to Ti^3+^ at relatively low reduction temperature (200 °C) in the presence of Pd. The dissociatively chemisorbed hydrogen on palladium may diffuse from Pd surface to TiO_2_ and reduce Ti^4+^ to Ti^3+^ ^38^.

The activity of Pd/Ⓕ-TiO_2_/AC catalyst for phenol hydrogenation was investigated. Corresponding results are listed in Table 1. The support Ⓕ-TiO_2_/AC showed no activity (Table 1, entry 1), whereas remarkable conversion and selectivity could be achieved with Pd loading on Ⓕ-TiO_2_/AC at 70 °C. When TiO_2_ loading was lower (Table 1, entry 2 and 3), phenol could not convert fully. When TiO_2_ loading was higher (Table 1, entry 4 and 5), the reaction stayed a conversion of exceeding 99.9 but slightly reduced the selectivity to cyclohexanone. With a fixed mole ratio of Pd and phenol, the conversion and selectivity remained about at the same, regardless of the loading of Pd (Table 1, entry 4, 6 and 7). The reaction was accelerated at higher temperature (Table 1, entry 6, 8–10). A fully conversion with a stable selectivity (around 97%) could be obtained in 1.5 h at 85 °C. It’s interesting that increasing the mole ratio of Pd and phenol could also accelerate the reaction (Table 1, entry 11 and 12). Notably, the mole ratio (0.5%) of Pd and phenol used here was rather lower than those in previous reports^17–21^, that is, the Pd/Ⓕ-TiO_2_/AC showed a higher efficiency than those reported catalysts to convert the same amount of phenol. The as-prepared complex nanostructured provide high dispersion and more contact interface with Pd nanoparticles. Thus, the outstanding performance of the as-prepared catalyst Pd/Ⓕ-TiO_2_/AC could be ascribed to the synergistic effect of metal nanoparticles and nano-semiconductor film layer, and the enhanced effect of electronic and the excellent charge transfer.

**Table 1:** Hydrogenation of phenol with Pd/Ⓕ-TiO_2_/AC catalyst under various conditions^a^.

Entry	Pd (wt. %)	TiO_2_ (wt.%)	T (°C)	Times (h)	Conversion (%)	Selectivity^b^ (%)	
						C=O	OH
1	0	10	70	5	—	—	—
2	2	0	70	2	66.6	96.0	4.0
3	1	5	70	2	86.9	97.1	2.9
4	1	10	70	2	>99.9	97.5	2.5
5	1	15	70	2	>99.9	96.7	3.3
6	2	10	70	2	>99.9	97.1	2.9
7	3	10	70	2	>99.9	96.7	3.3
8	2	10	40	10	>99.9	96.5	3.5
9	2	10	55	5	>99.9	96.7	3.3
10	2	10	85	1.5	>99.9	96.9	3.1
11^c^	2	10	70	0.5	93.0	98.6	1.4
12^d^	2	10	70	1	>99.9	97.8	2.2
13^e^	2	10	70	2	2.1	—	—

^a^Reaction conditions: phenol (0.353 g, 3.75 mmol), Pd (0.5 mol% relative to phenol), ichlomethane (20 mL), P*H*_2_ (5 bar), unless otherwise noted. ^b^C=O indicates cyclohexanone and C–OH indicates cyclohexanol. ^c^n_Pd_: n_phenol_ = 1.5%; ^d^n_Pd_ :n_phenol_ = 1%; ^e^substrate: cyclohexanone (0.368 g, 3.75 mmol).

To further understand the high selectivity, we used cyclohexanone as substrate to conduct hydrogenation experiment. The result showed that Pd/Ⓕ-TiO_2_/AC exhibited poor catalytic activity (Table 1, entry 13). Thus, the high hydrogenation selectivity of phenol to cyclohexanone could be attributed to the inhibition of cyclohexanone further hydrogenation to cyclohexanol by Pd/Ⓕ-TiO_2_/AC.

Generally, it is believed that the phenol hydrogenation proceeds under the cooperation between two types of activation work^22, 39^. One is the chemisorbed of phenol on the support, and the other is the activation of H_2_ on the adjacent Pd species. The adsorption orientation of phenol contains two modes: co-planar and non-planar. The former is formed by the aromatic ring connecting with the support surface, which tends to end up with the complete hydrogenation to cyclohexanol. The latter is formed by the oxygen atom linking with the support surface, which is favor of partial hydrogenation to cyclohexanone. Based on these results, we put forward the possible mechanism of phenol hydrogenation on Pd/Ⓕ-TiO_2_/AC (Figure S2). In the first step, most phenol is adsorbed on Ⓕ-TiO_2_/AC in non-planar orientation, slightly in co-planar orientation, because TiO_2_ is an amphiprotic oxide. Then, the adsorbed phenol is partially hydrogenated to cyclohexene by the activated hydrogen on the adjacent Pd nanoparticles. A co-planar adsorption orientation of cyclohexene could be easily hydrogenated to cyclohexanol because of the stronger chemisorption between the benzene ring and support, while the adsorbed cyclohexene in non-planar adsorption prefers to isomerize to give cyclohexanone quickly and leaves the surface of the catalyst rapidly because of only a weaker H-brige donor between the product and support.

Moreover, the electron-rich Pd nanopaticles could activate more H_2_ molecules. AC with abundant oxygen-containing group provide not only many electron, but also highly specific areas for the nanostructured of Pd and TiO_2_, which benefit mass transfer and the contact of reactants with active sites in phenol hydrogenation.

To further understand the synergistic effect of metal nanoparticles and nano-semiconductor film layer, we investigate the phenol hydrogenation over Pd/Ⓕ-CeO_2_/AC catalysts. (Table 2). As can be seen, the reaction reached completion in 1 hour and the selectivity attained >97% at 80 °C when 2%Pd/15%Ⓕ-CeO_2_/AC was used as catalyst (Table 2, entry 6). Interestingly, the conversion of phenol was low (77.5%) when the loading of CeO_2_ was 5%, while the conversion reached to >99% when CeO_2_ content was 10% or 15%. We believe these could be attribute to the nano-Ⓕ-CeO_2_ layer have not formed when the CeO_2_ loading was low so that the complex nanostructured of Pd/Ⓕ-CeO_2_ had not formed completely. Similar to Pd/Ⓕ-TiO_2_/AC, the phenol hydrogenation was accelerated at higher temperature over Pd/Ⓕ-CeO_2_/AC catalyst.

**Table 2:** Hydrogenation of phenol with Pd/Ⓕ-CeO_2_/AC catalyst under various conditions^a^.

Entry	Pd	CeO_2_	T(°C)	t(h)	Conversion (%)	Selectivity^b^ (%)	
						C=O	-OH
1	2	5	70	2	77.5	97.3	2.7
2	2	10	70	1.5	99.8	97.3	2.7
3	2	15	45	3	71.1	97.5	2.5
4	2	15	60	2.5	97.0	99.6	0.4
5	2	15	70	2	>99.9	95.6	4.4
6	2	15	80	1	>99.9	97.7	2.3

^a^Reaction conditions: phenol (0.353 g, 3.75 mmol), Pd (0.5 mol % relative to phenol), dichlomethane (20 mL), P*H*_2_ (5 bar), unless otherwise noted. ^b^C=O indicates cyclohexanone and C-OH indicates cyclohexanol.

To unveil the underlying factors that provide the superior activity of the hydrogenation over Pd/Ⓕ-MeO_x_/AC catalysts, we compared the TOFs for the hydrogenation of phenol over Pd/Ⓕ-MeO_x_/AC and other catalytic systems. As shown in Table 3, the results showed that Pd/Ⓕ-MeO_x_/AC catalysts gave a TOF of 200 h^−1^, an order of magnitude higher than traditional catalysts and 3.8-fold higher than that of the best reported catalyst PdAu/MHSS (ref. 15). It illustrated that the combination of nano-metal and nano-semiconductor, which both possess redox catalytic mechanism, was an efficient catalytic system and exhibited outstanding activity and selectivity for phenol hydrogenation.

**Table 3:** Comparison of different catalysts for phenol hydrogenation.

Entry	catalyst	n_Pd_:n_phenol_ (%)	T (°C)	time (h)	conv. (%)	sel. (%)		TOFs (h^−1^)^a^	Refs
						C=O	C-OH		
1	Pd/Ⓕ-TiO_2_/AC	0.5	70	2	>99.9	97.5	2.5	100	This work
2	Pd/Ⓕ-CeO_2_/AC	0.5	80	1	>99.9	97.7	2.3	200	This work
3	Pd/C-AlCl_3_	5	80	3	>99.9	99.3	0.7	6.67	17
4	Pd-mpg-C_3_N_4_	5	65	2	>99.9	>99	<1	10	18
5	Pd-PANI/CNT	5	80	9	>99.9	>99	<1	2.22	19
6	Pd-HPW	5	80	7	>99	>99	<1	2.86	21
7	PdAu/MHSS	2.5	50	0.75	97.5	96.6	3.4	52	15

^a^TOFs = mole of product/(moles of Pd * reaction times).

To fully illustrate the superiority catalytic performance of the complex nanostructured catalysts, we compared the catalytic results of the as-prepared Pd/Ⓕ-MeO_x_/AC, Pd/MeO_x_ and Pd/AC. As summarized in Table 4, Pd nanoparticles supported on the AC without metal oxide showed moderate activity for phenol hydrogenation (Table 4, entry 3). When Pd nanoparticles were supported on MeO_x_ (TiO_2_ or CeO_2_) simply without AC, the as-prepared samples had poor activity for phenol hydrogenation (Table 4, entries 4–5). Wang’s work^31^ also illustrated the poor hydrogenation over Pd/MeO_x_ catalysts (Table 4, entries 6–7). Nevertheless, catalysts Pd/Ⓕ-MeO_x_/AC, which were equipped with complex nanostructured of nano-Pd and nano-semiconductors film layer supported on the large surface support, exhibited predominant catalytic performance for phenol hydrogenation. In comparison with Pd/MeO_x_ catalysts, we could generalize two major structure features of the as-prepared Pd/Ⓕ-MeO_x_/AC: nano-metal and nano-semiconductor film layer supported on the large surface support and greatly increasing of contact interface of nano-metal-semiconductors. Owing to these excellent features, the properties of Pd/Ⓕ-MeO_x_/AC got improved: the synergistic effect of metal nanoparticles and nano-semiconductors support layer, the enhanced effect of electronic and the excellent charge transfer, and all these improvements may contribute to the enhanced catalytic performance.

**Table 4:** Catalytic results for Pd/Ⓕ-MeO_x_/AC, Pd/AC and Pd/MeO_x_ catalysts.

Entry	Catalysts	T(°C)	t(h)	Conversion (%)	Selectivity^a^ (%)		Reference
					C=O	C-OH	
1^b^	Pd/Ⓕ-TiO2/AC	70	2	>99.9	97.1	2.9	This work
2^b^	Pd/Ⓕ-CeO_2_/AC	70	1.5	99.8	97.3	2.7	This work
3^b^	Pd/AC	70	2	66.6	96.0	4.0	This work
4^b^	Pd/TiO_2_	70	2	6.4	99.4	0.6	This work
5^b^	Pd/CeO_2_	70	2	3.4	99.2	0.8	This work
6^c^	Pd/TiO_2_	65	6	8	99	1	40
7^c^	Pd/CeO_2_	65	6	2	100	0	40

^a^C=O indicates cyclohexanone and C-OH indicates cyclohexanol. ^b^Reaction conditions: phenol (0.353 g, 3.75 mmol), Pd (0.5 mol % relative to phenol), dichlomethane (20 mL), P_H2_ (5 bar). ^c^Reaction conditions: phenol 0.585 mmol, Pd (4 mol% relative to phenol), solvent 2 ml H_2_O, H_2_ 1 bar, reaction temperature 65 °C.

## Conclusion

In summary, a novel catalyst Pd/Ⓕ-MeO_x_/AC with complex nanostructured has been prepared by the photocatalytic reduction method. Palladium nanoparticles have been equably anchored on the nano-ⒻTiO_2_ film layer, which was spread on the high specific surface AC. Due to the complex nanostructured of metal nanoparticle and nano-semiconductors support layer, the as-prepared Pd catalyst Pd/Ⓕ-MeO_x_/AC exhibits a high activity for the phenol selective hydrogenation. The hydrogenation proceeds efficiently, with a lower mole ratio of Pd and phenol (0.5%) under the pressure of 0.7 MPa at 70 °C. The synergistic effect of metal nanoparticle and nano-semiconductors support layer, nano-metal-semiconductors heterojunction on the large surface support and greatly increasing of contact interface of nano-metal-semiconductors, the enhanced effect of electronic and the excellent charge transfer all could attribute to the outstanding catalytic performance. This work open a promising avenue for developing new supported metal catalyst with complex nanostructured of nano-Pd (Au, Pt, Rh, Ag, Ni, *et al*. metals) and nano-semiconductors film layer supported on the large surface support for efficient hydrogenation and redox reaction.

## Methods

### Synthesis

#### Pretreatment of activated carbon with acid

A commercial activated carbon (AC) made from coconut shells (Fujian Xinsen Carbon Co. Ltd.) was washed and boiled for 2–3 times. The activated carbon was pretreated with HNO_3_ (10%) at 60 °C under refluxing for 2 h, and eventually washed to neutrality with distilled water. Then the sample was dried at 80 °C for 12 h.

#### Preparation of Ⓕ-TiO_2_/AC support

The activated carbon was modified with 5, 10, 15 wt.% Ⓕ-TiO_2_ by sol-gel. Typically, 3 g tetra-n-butyl titanate was first dissolved in 25 mL ethanol by stirring and then 1.3 mL acetic acid was added into the mixture to obtain solution A. A solution of 15 mL ethanol and 1.5 mL distilled water was denoted by solution B. Then solution B was added into solution A dropwise under vigorous stirring to form sol C. Subsequently, the pretreatment AC was dispersed into sol C and nitric acid was used to adjust pH to 3. After being treated by ultrasonic agitation, the mixture was left at ambient temperature to form gel. The obtained material was dried at 80 °C for 12 h, and then calcined at 550 °C for 2 h. According to the principles of the spontaneous monolayer distribution, TiO_2_ loaded on the AC with monolayer or multilayers, rather than stacking pattern, by adjusting the loading of TiO_2_. The content of TiO_2_ was measured by ICP-AES analysis (Table S2). The as-prepared material was denoted as 10%Ⓕ-TiO_2_/AC.

#### Preparation of Pd-supported catalyst by the photocatalytic reduction method

The as-prepared TiO_2_/AC support (0.586 g) was dispersed in 100 mL distilled water and 3 mL methanol. Then the mixture was treated by ultrasonic agitation for 30 min. 1 mL of H_2_PdCl_4_ aqueous solution (0.0119 g/mL) was added into the slurry under stirring. The suspension was irradiated with a 15 W UV lamp for 10 h which is sufficient for deposition of palladium at ambient temperature. Finally, the sample was separated by filtration, washed several times by distilled water up to pH 7, dried in a vacuum oven for 10 h. This sample will be referred as Pd/Ⓕ-TiO_2_/AC. The load of Pd was measured by ICP-AES analysis and the results showed that the loading efficiency of Pd exceeded 98% (Table S2). Here, we use the theory content of Pd to describe the as-prepared catalysts. The principle of photocatalytic reduction method could been summarized as following:

1 $${{\rm{TiO}}}_{2}+{\rm{hv}}\to {{\rm{TiO}}}_{2}({{\rm{e}}}^{-}+{{\rm{h}}}^{+})$$

2 $${{\rm{Pd}}}^{2+}+2{{\rm{e}}}^{-}\to {\rm{Pd}}$$

3 $${{\rm{O}}}_{2}+4{{\rm{H}}}^{+}+4{{\rm{e}}}^{-}\to 2{{\rm{H}}}_{2}{\rm{O}}$$

4 $$2{{\rm{H}}}_{2}{\rm{O}}+4{{\rm{h}}}^{+}\to {{\rm{O}}}_{2}+4{{\rm{H}}}^{+}$$

5 $${\rm{Total}}\,{\rm{reaction}}:2{{\rm{Pd}}}^{2+}+2{{\rm{H}}}_{2}{\rm{O}}\,\mathop{\to }\limits^{{\rm{hv}}}2{\rm{Pd}}+{{\rm{O}}}_{2}+4{{\rm{H}}}^{+}$$

#### Phenol Hydrogenation

Hydrogenation of phenol was performed in a 50 mL Teflon-lined stainless steel autoclave. Typically, 0.353 g phenol, a given amount of Pd catalyst (Pd/phenol = 0.005, 0.01, 0.015 (ratio of mole)) and 20 mL dichloromethane were loaded into the reactor. The autoclave was sealed and purged three times with H_2_ to remove the air. Then the reactor was placed in an oil bath. Hydrogen was introduced into the reactor after desired temperature was reached and the stirrer was started. After the reaction, the reactor was cooled to room temperature and the products were analyzed on GC (Figure S3).

The conversion and selectivity are defined as follows:

6 $${\rm{Conversion}}\,{\rm{of}}\,\mathrm{phenol}\,( \% )=\,\frac{{\rm{Moles}}\,{\rm{of}}\,{\rm{phenol}}\,{\rm{converted}}}{{\rm{Moles}}\,{\rm{of}}\,{\rm{phenol}}\,{\rm{initially}}\,{\rm{added}}}\ast 100 \% $$

7 $${\rm{Selectivity}}\,{\rm{to}}\,\mathrm{cyclohexanone}\,( \% )=\,\frac{{\rm{Moles}}\,{\rm{of}}\,{\rm{cyclohexanone}}}{{\rm{Moles}}\,{\rm{of}}\,{\rm{phenol}}\,{\rm{converted}}}\ast 100 \% $$

#### Characterization

X-ray diffraction (XRD) of samples was obtained on a Rigaku D/max-II/2500 X-ray powder diffractometer, Cu Ka radiation was employed and the working voltage and current were 40 kV and 30 mA, respectively. Surface images of the samples were investigated by a JEM2010 transmission electron microscopy (TEM). High resolution transmission electron microscopy (HRTEM) images were obtained using a JEOL JEM-2100F transmission electron microscope at an acceleration voltage of 200 kV. Fourier transform infrared (FT-IR) spectra were recorded by using a Nicolet 380 spectrometer from 4000 to 400 cm^−1^, and the KBr pellet technique was used. X-ray photoelectron spectroscopy (XPS) was performing using ESCALAB 250Xi (Thermo) with Al Kα radiation. Inductively coupled plasma-atomic emission spectrometry (ICP-AES) data were obtained using an Intrepid II XSP (IRIS). H_2_ temperature programmed reduction (H_2_-TPR) were carried out on ChemBET 3000 with a thermal conductivity detector. The samples were treated at 250 °C for 1 h under Ar atmosphere, then cooled down to 80 °C. After pre-treated, temperature was increased to 700 °C at the heating rate of 10 °C/min in a 10% H_2_/Ar stream.

## Electronic supplementary material

Novel nano-semiconductor film layer supported nano-Pd Complex Nanostructured Catalyst Pd/Ⓕ-MeOx/AC for High Efficient Selective Hydrogenation of Phenol to Cyclohexanone
