# Supplementary material for: Novel nano-semiconductor film layer supported nano-Pd Complex Nanostructured Catalyst Pd/Ⓕ-MeOx/AC for High Efficient Selective Hydrogenation of Phenol to Cyclohexanone
Source: Sci Rep. 2017 Apr 28;7:1254. doi: 10.1038/s41598-017-01255-9 (PMC5430673; doi:10.1038/s41598-017-01255-9)
Supplement: Supplementary file 1 — Novel nano-semiconductor film layer supported nano-Pd Complex Nanostructured Catalyst Pd/Ⓕ-MeOx/AC for High Efficient Selective Hydrogenation of Phenol to Cyclohexanone [file 41598_2017_1255_MOESM1_ESM.doc]

**Supporting information**

**Novel nano-semiconductor film layer supported nano-Pd Complex Nanostructure Catalyst Pd/ⒻMeO_x_/AC for High Efficient Selective Hydrogenation of Phenol to Cyclohexanone**

Jiaqi Si^1^, Wenbing Ouyang^1^, Yanji Zhang^1^, Wentao Xu^1^ and Jicheng Zhou^1^.

^1^Key Laboratory of Green Catalysis and Chemical Reaction Engineering of Hunan Province, School of Chemical Engineering Xiangtan University, Xiangtan 411105, Hunan Province, China.

Correspondence and requests for material should be addressed to Jicheng Zhou (**email:**  [zhoujicheng@sohu.com](mailto:zhoujicheng@sohu.com))

**Table S1** Surface area and pore structure of the samples.

| Samples | BET  Surface area (m^2^/g) | BJH desorption summary | | |
| --- | --- | --- | --- | --- |
|  |  | Surface area (m^2^/g) | Pore volume (cc/g) | Pore diameter (nm) |
| AC | 1389 | 428 | 0.332 | 1.698 |
| 10%Ⓕ-TiO_2_/AC | 1241 | 417 | 0.329 | 1.692 |
| 2%Pd/10%Ⓕ-TiO_2_/AC | 1138 | 376 | 0.291 | 1.676 |

**TableS2.** ICP-AES of the as-prepared Pd/x%Ⓕ-TiO_2_/AC catalysts

| Entry | Nominal TiO_2_ (wt.%) | Actual TiO_2_ (wt.%) |
| --- | --- | --- |
| 1 | 5 | 5.24 |
| 2 | 10 | 10.66 |
| 3 | 15 | 15.48 |

**TableS3.** ICP-AES of the as-prepared Pd catalysts

| Entry | Nominal Pd (wt.%) | Actual Pd (wt.%) | Loading efficiency^a^ (%) |
| --- | --- | --- | --- |
| 1 | 1.0 | 0.987 | 98.7% |
| 2 | 1.5 | 1.489 | 99.3% |
| 3 | 2.0 | 1.968 | 98.4% |

^a^ Loading efficiency = actual Pd/Nominal Pd


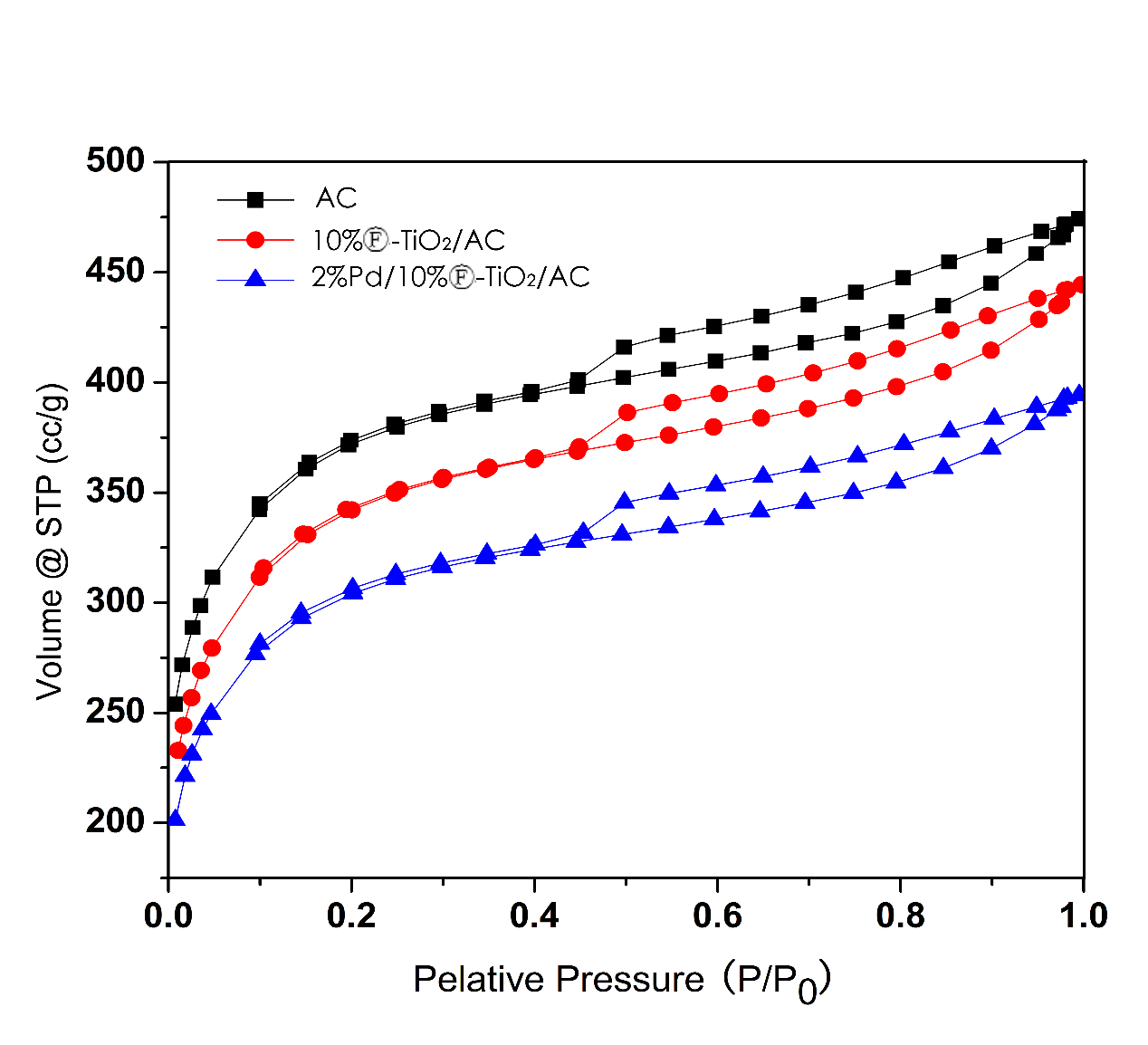


**Figure S1** N_2_ adsorption/isotherms of AC, 10%Ⓕ-TiO_2_/AC and 2%Pd/10%Ⓕ-TiO_2_/AC.


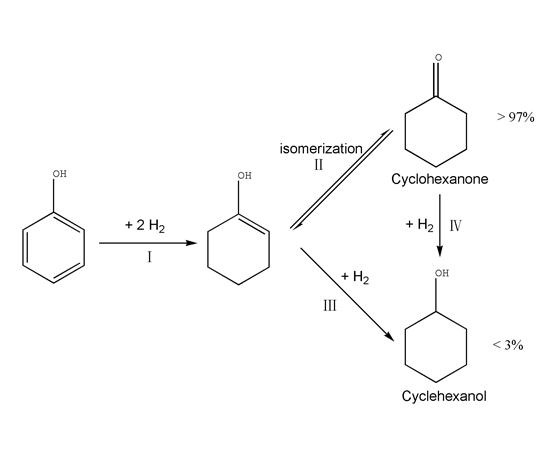


**Figure S2.** Possible Reaction Mechanism of Phenol over Pd/Ⓕ-TiO_2_/AC


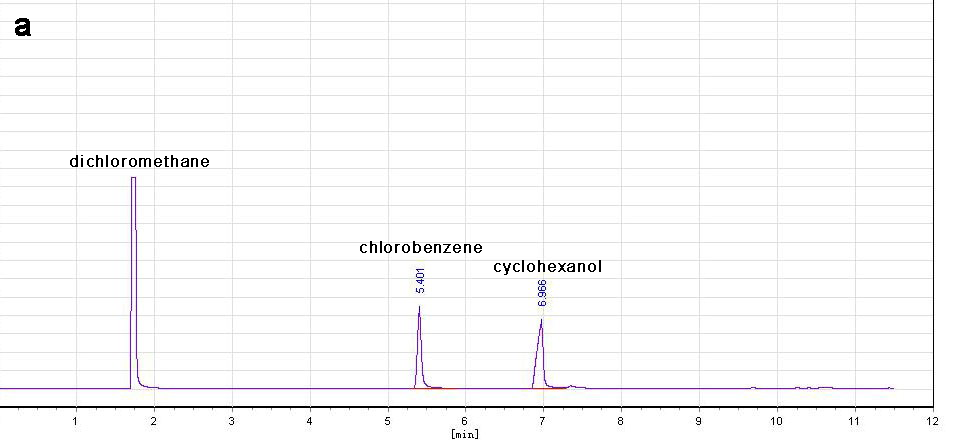


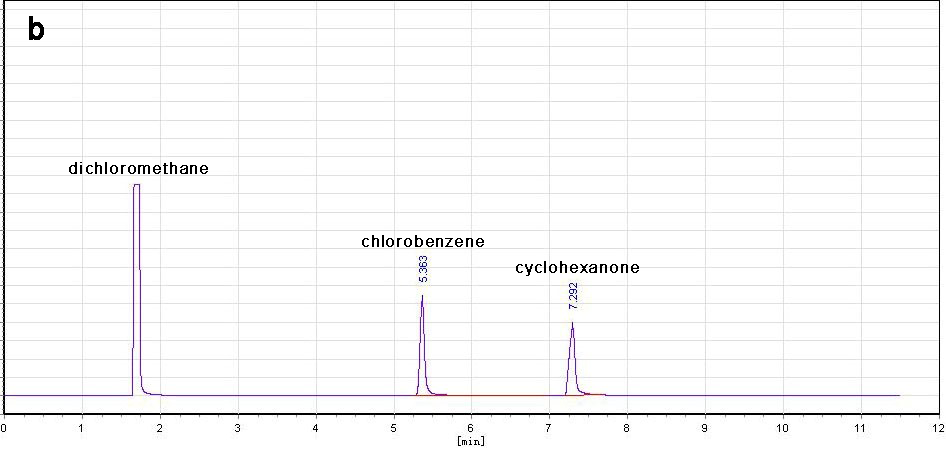


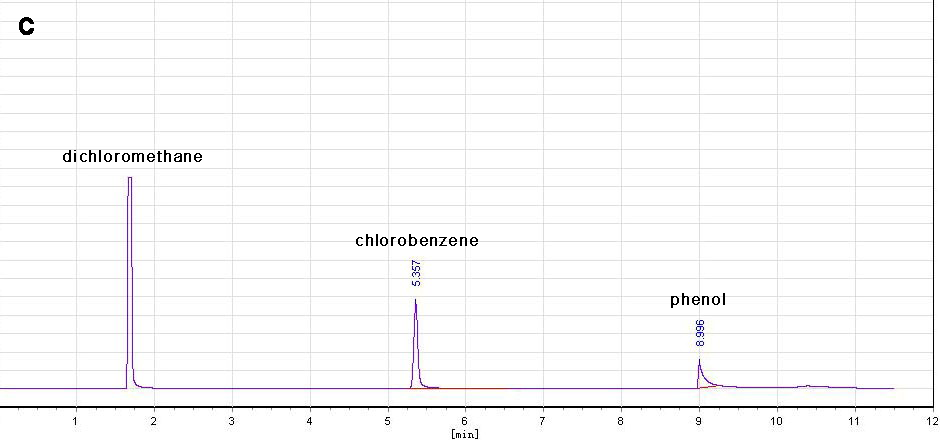

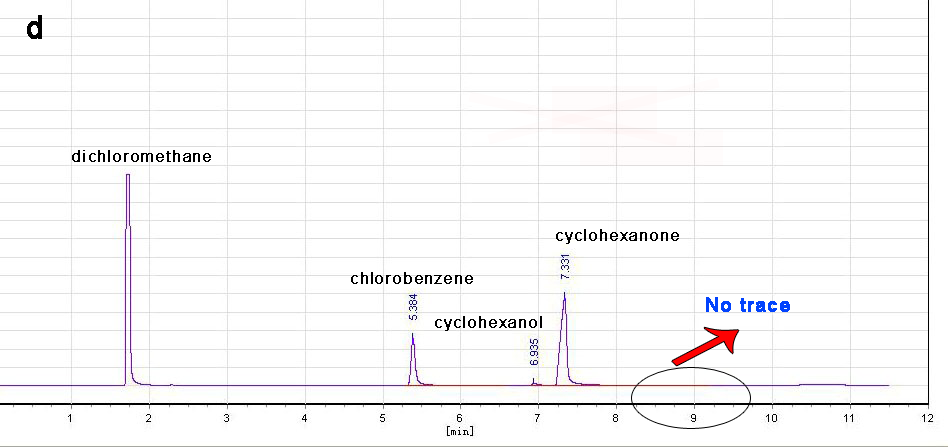


**Figure S3**. The GC traces of the samples: (a) cyclohexanol; (b) cyclohexanone; (c) phenol; (d) Table 1 entry 6.
